# Lower Back Pain Imaging: A Readability Analysis

**DOI:** 10.7759/cureus.45174

**Published:** 2023-09-13

**Authors:** Michael J Valentine, Gannon Cottone, Hunter D Kramer, Ankur Kayastha, James Kim, Nicholas J Pettinelli, Robert C Kramer

**Affiliations:** 1 College of Osteopathic Medicine, Kansas City University, Kansas City, USA; 2 Medicine, Kansas City University, Kansas City, USA; 3 Hand Surgery, Beaumont Bone and Joint Institute, Beaumont, USA

**Keywords:** click-through rate, lumbar pain, radiography, lower back pain, back pain, online information, radiograph, imaging, readability, health literacy

## Abstract

Purpose: The internet provides access to a myriad of educational health-related resources which are an invaluable source of information for patients. Lower back pain is a common complaint that is discussed extensively online. In this article, we aim to determine if the most commonly accessed articles about lower back pain imaging use language that can be understood by most patients. According to the American Medical Association (AMA) and National Institute of Health (NIH), this corresponds to a sixth-grade reading level.

Methods: Online searches were conducted from the most commonly used search engine, Google, to assess the present state of readability on radiograph imaging for LBP. Then the top 20 populated URL links from each search were utilized based on “health & fitness” search trends and click-through rates (CTRs). The readability of various websites was evaluated with WebFX online software that analyzed the unique websites' text when put into reader view on Firefox web browser version 116.0.3 (64-bit). Evaluation occurred via five common readability indices: the Automated Readability Index (ARI), the Coleman Liau Index (CLI), the SMOG index, the Gunning Fog Score Index (GFSI), and the Flesch Kincaid Grade Level Index (FKGLI). In addition, the Flesch Kincaid Reading Ease Index (FKREI) was also used but was excluded from the calculation due to its measuring scale outside of US grade levels. The number of samples was analyzed via health and fitness-specific CTR from an open-access database from July 2022 to July 2023. This was used to calculate the number of persons clicking and visiting positional URLs (first URL to the 20th URL) from each unique keyword search and the rational criteria for selecting the first 20 websites for each query.

Results: Online material that included LBP imaging information was calculated to have an overall readability score of 10.745 out of the 23 websites obtained from unique searches. The range was a mean readability score of 8 to 14. Notably, 17 websites were excluded from a total of 40 websites due to duplication of the same data (URLs that resulted from both unique searches) and accessibility requiring payment (specifically, an UpToDate link). A readability score of 10.745 refers to an 11th-grade reading level. That is to say, the most commonly visited sites on Google that contain information about lower back pain imaging are, on average, five grade levels higher than the sixth-grade reading level recommended by the AMA and the NIH.

Conclusions: Most internet content regarding lower back pain imaging is written at a reading level that is above the recommended limit defined by the AMA and NIH. To improve education about lower back pain imaging and the patient-physician relationship, we recommend guiding patients to online material that contains a reading level at the sixth-grade level as suggested by the AMA and NIH.

## Introduction

When determining the etiology of a patient’s acute lower back pain (LBP), a physician may or may not choose to order radiographic imaging. The necessity to incorporate imaging in the initial assessment of LBP depends on a variety of factors, such as the duration of pain, presence of red-flag symptoms, patient age, history of trauma, and prior history of back pain [1]. However, for patients experiencing acute LBP - defined as pain persisting for less than four weeks - without any accompanying symptoms, the utility of obtaining imaging is considered quite low and has been associated with increased invasive procedures and health care costs [2]. In addition, radiation exposure has the capacity to cause uncontrolled chemistry within the body that may result in potential harm. Therefore, it is appropriate to forgo imagining in the initial evaluation of low-risk LBP patients.

This rationale, albeit grounded in evidence, may elicit apprehension among patients desiring a conclusive explanation for their discomfort. In instances where a physician’s proposed care plan and the patient’s expectations are incongruent, it is imperative for the physician to elucidate the evidence guiding their decision-making rationale. While this can often be achieved in an in-person consultation, physicians often face time constraints during patient interactions. The risk-benefit analysis implicated in the decision to order radiographic imaging is intricate and necessitates a detailed explanation. Fortunately, there are many online resources available that may illuminate this topic for patients. These resources constitute a valuable asset for busy physicians who aim to provide their patients with comprehensive education.

The abundance of health information online is an invaluable feature of our modern world and can significantly aid the process of patient education. However, the quality of resources exhibits considerable variation. While it is clear that each resource should be evaluated for quality before recommending it to a patient, readability is an overlooked measure that is less frequently scrutinized. Although numerous articles are written for the purpose of patient education, our analysis reveals that the language employed in the most widely accessed articles is too complex for the average patient. To ensure the utility of these resources for the majority of patients, it is essential that they are composed of language accessible to individuals with a reading proficiency equivalent to the sixth grade. This analysis sheds light on this discrepancy and encourages physicians to evaluate the language of online resources before sharing them with their patients.

## Materials and methods

Readability analysis

Several objective indices have been employed to assess the readability of online text pertaining to various subjects. These indices, characterized by different mathematical formulas, focus on varied facets of textual parameters. This study incorporates six of the most common indices surveyed from literature: the Flesch Kincaid reading ease (FKRE) test, the Flesch Kincaid grade level (FKGL) test, the Gunning Fog score (GFS), the Coleman Liau index (CLI), the Automated Readability Index (ARI), and the Simple Measure of Gobbledygook (SMOG) index. These indices are commonly used for readability analysis [3-10]. Excluding FKRE, the FKGL, GFS, CLI, ARI, and SMOG indices all estimate the minimum US grade level required to understand a text, albeit each index incorporates its unique syntactical characteristic. These indices were calculated by using the WebFX readability tool.

The FKRE calculates scores ranging from 1 to 100, denoting higher readability with a score that is closer to 100. Due to its distinct scoring, FKRE was excluded from readability analysis with the other indices but was still averaged. FKGL, on the other hand, yields the US grade level for a given text. For example, a FKGL score of 7 implies that comprehension of a given text may be understood by a seventh grader. Notably, both FKRE and FKGL's formulas are derived from sentence length and word length.

Conversely, the GFS index approximates the amount of educational years a person requires to comprehend a given text. Similar to the FKGL, the calculated score denotes the US grade level required for understanding. However, this index characterizes the complexity of words by categorizing complex words as words with three or more syllables.

The CLI is a readability test designed to measure the understandability of a text. This index theorizes that word length is a better predictor of readability than syllables. The ARI is similar but instead of counting syllables, the ARI counts characters and sentences. The more characters and sentences a given text has, the more difficult the word and text are theorized to become. Finally, the SMOG index contains the simplest formula and is useful for the clarity of a text’s message. Given that no single index is considered superior to the others, the total average of all indices was calculated in an effort to determine a more accurate readability score.

Keyword rational

Initially, foundational topics encompassing “low back pain,” “lumbar spine,” “radiograph,” and “imaging” were delineated. Subsequent to this, keywords were selected in alignment with these identified themes. Recognizing the inherent variability in subjective patient search queries, the focus was directed toward objective searches based on the overarching topic rather than predicting individualized terms that patients might employ. From this process, two phrases were selected: “low back pain x-ray” and “imaging for lower back pain.” Considering that a predominant portion of internet searches in the US are conducted via Google, this platform was chosen as the designated targeted search engine for this study [11].

Readability analysis and data collection

An examination of the readability of online information related to LBP imaging was conducted using Google on August 17, 2023, since Google contains 81% of the US search engine market [11]. For each unique keyword search query, the initial 20 results from the search engine's results page were incorporated into the study. However, advertisements, sponsored links, and videos were systematically excluded from the data compilation. Utilizing the Firefox web browser (version 116.0.3) (64-bit), the “Reader mode” was activated for each accessed URL. Subsequently, all text content displayed within this mode was extracted and transposed into the input field of the WebFx Readability Test Tool [12]. This tool provided outputs based on six distinct readability indices. The resultant data were then cataloged and analyzed using Google Sheets, with data organized according to its individual readability index score (Table 1).

**Table 1:** Websites utilized for readability analysis for two different search queries: “low back pain x-ray” and “imaging for low back pain.” ^1^ Website excluded due to accessibility requiring payment (specifically, an UpToDate). All bolded URLs were excluded from a total of 40 websites due to duplication (URLs that resulted from both unique searches).

Position in Query	Google search #1: "low back pain x-ray"	Google search #2: "imaging for lower back pain"
1	https://www.ncbi.nlm.nih.gov/pmc/articles/PMC2697333/	https://www.ncbi.nlm.nih.gov/pmc/articles/PMC7529135/
2	https://www.aafp.org/family-physician/patient-care/clinical-recommendations/all-clinical-recommendations/cw-back-pain.html	https://www.aafp.org/family-physician/patient-care/clinical-recommendations/all-clinical-recommendations/cw-back-pain.html
3	https://www.healthcentral.com/condition/back-pain/low-back-pain/do-really-need-ray-or-mri-lower-back-pain	https://www.ncbi.nlm.nih.gov/pmc/articles/PMC6118107/
4	https://www.ucsfhealth.org/medical-tests/lumbosacral-spine-x-ray	https://choosingwiselycanada.org/pamphlet/imaging-tests-for-lower-back-pain/
5	https://www.hopkinsmedicine.org/health/treatment-tests-and-therapies/xrays-of-the-spine-neck-or-back	https://www.jospt.org/doi/10.2519/jospt.2011.3618
6	https://www.webmd.com/back-pain/spinal-x-ray-overview	https://www.uptodate.com/contents/evaluation-of-low-back-pain-in-adults/print?search=Low%20back%20pain%20considerations%20for%20imaging&source=search_result&selectedTitle=3~150&usage_type=default&display_rank=21
7	https://choosingwiselycanada.org/pamphlet/imaging-tests-for-lower-back-pain/	https://www.ncqa.org/hedis/measures/use-of-imaging-studies-for-low-back-pain/
8	https://burlingtonsportstherapy.com/blog/do-i-need-an-x-ray-for-back-pain/	https://www.bmj.com/content/372/bmj.n291
9	https://www.healthline.com/health/lumbosacral-spine-x-ray	https://journalofethics.ama-assn.org/article/imaging-modalities-back-pain/2007-02
10	https://www.s-spinehospital.com/en/x-ray-or-mri/	https://www.envrad.com/imaging-tests-used-for-diagnosing-back-pain/
11	https://www.mountsinai.org/health-library/tests/lumbosacral-spine-x-ray	https://www.hss.edu/conditions_imaging-back-pain.asp
12	https://www.ncqa.org/hedis/measures/use-of-imaging-studies-for-low-back-pain//	https://medlineplus.gov/ency/article/007493.htm
13	https://www.labfinder.com/radiology/xray/x-ray-back-lumbar-spine-lower-back/	https://acsearch.acr.org/docs/69483/narrative/
14	https://myteampt.com/low-back-pain-do-i-need-an-mri-or-x-ray-before-seeing-a-physical-therapist/	https://www.healthcentral.com/condition/back-pain/low-back-pain/do-really-need-ray-or-mri-lower-back-pain
15	https://www.osteopathyabingdon.co.uk/general-interest/i-have-back-pain-should-i-have-an-x-ray/	https://www.racgp.org.au/getattachment/f9bafb24-786d-44db-8cf9-333ec2c5245f/attachment.aspx
16	https://www.envrad.com/imaging-tests-used-for-diagnosing-back-pain/	https://www.acpjournals.org/doi/10.7326/0003-4819-154-3-201102010-00008
17	https://www.acpjournals.org/doi/10.7326/0003-4819-154-3-201102010-00008	https://www.ajronline.org/doi/full/10.2214/AJR.10.4367
18	https://www.sahealth.sa.gov.au/wps/wcm/connect/public+content/sa+health+internet/clinical+resources/clinical+programs+and+practice+guidelines/medical+conditions/orthopaedics/lumbar+disorders/clinical+resources+for+lumbar+disorders/spinal+imaging+recommendations	https://www.sahealth.sa.gov.au/wps/wcm/connect/public+content/sa+health+internet/clinical+resources/clinical+programs+and+practice+guidelines/medical+conditions/orthopaedics/lumbar+disorders/clinical+resources+for+lumbar+disorders/spinal+imaging+recommendations
19	https://acsearch.acr.org/docs/69483/narrative/	https://www.intechopen.com/chapters/70568
20	https://www.brevarddisc.com/diagnosis-of-low-back-pain.html	https://www.thelancet.com/journals/lancet/article/PIIS0140-6736(09)60172-0/fulltext

Click-through rate

CTR is calculated by the number of times a link is clicked divided by the number of times the link is shown on a search engine results page. Another way of framing CTR is to conceptualize CTR as a means of measuring online traffic to websites. One year of “health & fitness” specific CTR data was acquired via an open-access database from July 2022 to July 2023, as well as four years of data from July 2019 to July 2023 (Table 2) [12,13]. The rationale for analyzing the top 20 websites per unique query was based on the resulting sums of “health & fitness” specific CTR data.

**Table 2:** Click-through rate (CTR) data generated looking at both one-year (July 2022-July 2023) and four-year (July 2019-July 2023) intervals. ^1^ Sum of top 3, top 10, & top 20 google search positions. Total percentages for the top 3, 10, and 20 search positions are also noted (bold). Abbreviations: US - United States, Int'l - International, CTR - Click Through Rate.

	1 Year				4 Year			
SERP Position	US Predicted Click % - 1y	Sum of US CTR - 1y	Int'l Predicted Click % - 1y	Sum of Int'l CTR - 1y	US Predicted Click % - 4y	Sum of US CTR - 4y	Int'l Predicted Click % - 4y	Sum of Int'l CTR - 4y
1	19.24	-	22.44	-	21.44	-	25.14	-
2	12.49	-	14.20	-	14.57	-	16.31	-
3	7.61	^1^ 39.34%	9.02	^1^ 45.67%	9.38	^1^ 45.38%	10.76	^1^ 52.21%
4	4.82	-	5.75	-	6.29	-	7.35	-
5	3.15	-	3.93	-	4.30	-	5.15	-
6	2.19	-	2.85	-	2.98	-	3.73	-
7	1.61	-	2.16	-	2.14	-	2.83	-
8	1.22	-	1.69	-	1.62	-	2.18	-
9	0.96	-	1.35	-	1.24	-	1.72	-
10	0.80	^1^ 54.10%	1.16	^1^ 64.57%	0.98	^1^ 64.94%	1.40	^1^ 76.57%
11	0.78	-	1.12	-	0.83	-	1.22	-
12	0.84	-	1.17	-	0.79	-	1.18	-
13	0.89	-	1.20	-	0.86	-	1.23	-
14	0.94	-	1.19	-	0.94	-	1.25	-
15	0.97	-	1.16	-	1.03	-	1.24	-
16	0.98	-	1.10	-	1.11	-	1.21	-
17	0.94	-	1.03	-	1.16	-	1.16	-
18	0.89	-	0.97	-	1.17	-	1.10	-
19	0.85	-	0.92	-	1.10	-	1.04	-
20	0.78	^1^ 62.98%	0.86	^1^ 75.29%	1.03	^1^ 74.94%	0.98	^1^ 88.17%

## Results

CTR results in the United States (US) from July 2022 to July 2023 demonstrated that the top 20 links covered over 62.98% of searches. Over four years, 74.94% were covered. The annual CTR internationally for the same time frames was 74.94% and 88.17%, respectively (Table 2).

**Figure 1:**
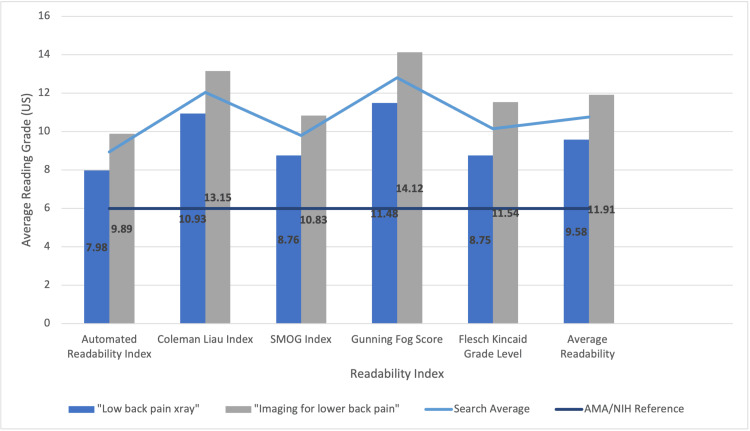
Readability results for searching both “low back pain x-ray” and “imaging for lower back pain,” along with a sixth-grade reading reference line (dark blue) and search averages (light blue line). Abbreviations: AMA - American Medical Association, NIH - National Institutes of Health

In our evaluation of 40 websites focusing on the readability of radiographs for low back pain, 16 (eight websites for each search query) were omitted due to duplication that would have resulted in the same readability score. Additionally, the website "UpToDate" was excluded given its restricted access. When analyzing search results for "lower back pain x-ray," the mean readability score was found to be 9.58 (Table 3) [12]. In contrast, the search term "Imaging for lower back pain" resulted in a higher mean readability score of 11.91 (Table 3). This discrepancy highlights a more difficult readability level when including “imaging” within the unique search query. The cumulative mean readability score (“lower back pain x-ray” search and “imaging for lower back pain” search) yielded a score of 10.745 (Table 3). This suggests that the predominant online content is comprehensible at roughly the 11th-grade US level. This is approximately five grade levels beyond the sixth-grade threshold endorsed by the American Medical Association (AMA) and National Institutes of Health (NIH) (Figure 1).

**Table 3:** Average readability from WebFX that includes two searches “low back pain x-ray” and “imaging for lower back pain” as well as the total readability average across search terms. 1 Flesch Kincaid Reading Ease Index (FKREI) was excluded from the calculation and average readability due to a different grade scale. Abbreviations: SMOG - Simple Measure of Gobbledygook

	"Low back pain xray"	"Imaging for lower back pain"	Search Average
Automated Readability Index	7.98	9.89	8.94
Coleman Liau Index	10.93	13.15	12.04
SMOG Index	8.76	10.83	9.80
Gunning Fog Score	11.48	14.12	12.80
Flesch Kincaid Grade Level	8.75	11.54	10.15
Flesch Kincaid Reading Ease	^1^ 60.98	^1^ 41.27	^1^ 51.13
Average Readability	9.58	11.91	10.75

## Discussion

A considerable proportion of the adult population will experience LBP at some point in their life. Studies have found that LBP is the leading worldwide cause of disability [13,14]. Economically, LBP poses a significant burden. Direct and indirect costs in the US are approximated to be $50 billion annually in one study [15]. Another study estimated total healthcare-related expenses for LBP in the US to be $87.6 billion [16].

Given the widespread prevalence of LBP, there has been an overabundance of internet content pertaining to this symptom, including guidelines on when imaging is warranted. Internet resources have quickly become the dominant method by which patients obtain information regarding their health. Consequently, it is of paramount importance that the most accessible online articles not only contain accurate information but are also comprehensible to the general public.

In 2022, the National Literacy Institute reported that approximately 21% of Americans were illiterate, with an additional finding that 130 million adults lacked the capability to read a bedtime story to their children. Furthermore, it was found that the literacy level of 54% of American adults was below that of a sixth grader [17]. Unfortunately, a significant portion of online health content is not only deficient in quality but is also excessively dense [18,19]. Our analysis demonstrates that the complexity of online LBP imaging information corresponds to approximately an 11th-grade reading level (Table 3). This implies that a considerable segment of the patient population may find the information in these articles inaccessible, thereby limiting their access to valuable information and consequently reducing their healthcare literacy. 

Skilled physicians often educate their patients to clarify treatment rationale. However, due to constrained consultation limitations, patients are often sent home with educational material for review. Beyond physical handouts, it is critical to guide patients toward appropriate online resources, anticipating their online inquiries about LBP. Therefore, it is crucial that professional healthcare workers are aware of where to find accurate, trustworthy data that is distilled into a sixth-grade reading level before the patient encounter. Coinciding with the trend of physicians ordering imaging on non-specific LBP and theorized patient-driven factors, it may be possible that this advanced readability level could inadvertently promote the overuse of routine LBP imaging. However, stronger research is warranted to explore this association.

Limitations

This readability analysis is subject to limitations. Firstly, search results will change with time. Secondly, the study did not include images, sponsored websites generated by Google, or videos. Thirdly, there was a notable overlap between the URLs that were generated from each search phrase resulting in duplication of websites (Table 1). Due to the consistent readability outcomes from this overlap, a decision was made to exclude eight redundant URLs (16 websites of the generated total of 40 websites). Furthermore, one URL was excluded that required access in order to read (an UpToDate link). Notwithstanding, this third set of limitations does not seem to have a considerable diminishing effect considering the mean readability score for “lower back pain x-ray” yielded 9.58 and the mean readability score for “imaging for lower back pain” yielded 11.91 (Figure 2). Lastly, in reference to the chosen keywords, it would have been more advantageous to gather a questionnaire from a sample group to more accurately determine frequently searched keywords.

## Conclusions

The readability of online information pertaining to LBP imaging was determined to average a score of 10.75, which correlates to the reading proficiency expected of an 11th-grade student in the US. Notably, this is five grade levels higher than the AMA and NIH recommended guidelines. As this diagnosis is extremely common among adults, it is critical that the most popular online resources regarding the subject are accurate and written for a general audience. Access to accurate information regarding a particular diagnosis is critical for patients who hope to understand it and will facilitate the health literacy of our patients. In this paper, we hope to highlight that much of the health information online regarding the indications for imaging in LBP may be written in a way that cannot be easily understood by a large number of Americans and therefore is inaccessible to them. We urge physicians to evaluate the online resources that they frequently recommend for ease of readability and encourage the publication of health resources targeted at a more general audience.
